# A Statistical Note on an Analysis of Cases of Tetanus in the British Expeditionary Forces

**Published:** 1918-01

**Authors:** 


***A Statistical Note on an Analysis of Cases of Tetanus in the British Expeditionary Forces.*** By M. GREENWOOD, Jr. Capt. R. A. M. C. (T. F.). Abs. from the *Lancet*, May 5, 1917.

G., who is a medical statistician, objects strongly to the presumption against the intrathecal route of antitoxin therapy which Leishman and Smallman believe they have established from the statistics analysed by them (see p. 189). He thinks their data far too meager to make possible any accurate conclusions on the point in question. Such variations as appear to favor their assumption, he thinks, may easily be accounted for by the element of chance, against which the statistician must always be on his guard.

G. reexamines the statistics given by L. and S. with a view to determining whether the variations in the case mortalities of the 15 groups studied by these authors are greater than might be attributed to chance. G. accordingly tabulates the statistics of L. and S. in order to compare them with the normally expected results in a uniform population. G. concludes that the variations are no more than might be expected, and, therefore, that L. and S. have created no presumption at all against the value of the intrathecal method of administering tetanus antitoxin.

